# A Genetic Algorithm Based Support Vector Machine Model for Blood-Brain Barrier Penetration Prediction

**DOI:** 10.1155/2015/292683

**Published:** 2015-10-04

**Authors:** Daqing Zhang, Jianfeng Xiao, Nannan Zhou, Mingyue Zheng, Xiaomin Luo, Hualiang Jiang, Kaixian Chen

**Affiliations:** ^1^Center for Systems Biology, Soochow University, Suzhou 215006, China; ^2^Drug Discovery and Design Center, State Key Laboratory of Drug Research, Shanghai Institute of Materia Medica, Chinese Academy of Sciences, 555 Zuchongzhi Road, Shanghai 201203, China; ^3^School of Pharmacy, East China University of Science and Technology, Shanghai 200237, China

## Abstract

Blood-brain barrier (BBB) is a highly complex physical barrier determining what substances are allowed to enter the brain. Support vector machine (SVM) is a kernel-based machine learning method that is widely used in QSAR study. For a successful SVM model, the kernel parameters for SVM and feature subset selection are the most important factors affecting prediction accuracy. In most studies, they are treated as two independent problems, but it has been proven that they could affect each other. We designed and implemented genetic algorithm (GA) to optimize kernel parameters and feature subset selection for SVM regression and applied it to the BBB penetration prediction. The results show that our GA/SVM model is more accurate than other currently available log *BB* models. Therefore, to optimize both SVM parameters and feature subset simultaneously with genetic algorithm is a better approach than other methods that treat the two problems separately. Analysis of our log *BB* model suggests that carboxylic acid group, polar surface area (PSA)/hydrogen-bonding ability, lipophilicity, and molecular charge play important role in BBB penetration. Among those properties relevant to BBB penetration, lipophilicity could enhance the BBB penetration while all the others are negatively correlated with BBB penetration.

## 1. Introduction

The blood-brain barrier (BBB) plays important roles in separating the central nervous system (CNS) from circulating blood and maintaining brain homeostasis. BBB penetration, which may be desired or not depending on the therapeutic target, is a critical character in chemical toxicological studies and in drug design. Compounds can cross the BBB by passive diffusion or by means of a variety of catalyzed transport systems that can carry compounds into the brain (carrier-mediated transport, receptor-mediated transcytosis) or out of the brain (active efflux). Various parameters are used for predicting BBB penetration such as CNS+/−, log⁡*BB*, and log⁡*PS*. CNS+/− is a qualitative property denoting the compound's activity (CNS+) or inactivity (CNS−) against a CNS target with its BBB penetration [1]. The problem with CNS+/− datasets is that CNS activity implies BBB permeation, while CNS inactivity might be due to factors other than nonpermeation, such as the fact that compounds might be rapidly metabolized or effluxed from the brain. Log *BB*, which is defined as logarithm of Brain/Blood partitioning ratio at steady state [2], is by far the most widely used parameter for BBB penetration. However, this parameter may also result in misleading conclusions because it ignores the main parts of process of permeability [3]. Log* PS*, which is defined as the logarithm of permeability-surface area product reflecting the rate of brain permeation, is superior to but more difficult to measure compared to log⁡*BB* [4].* In vivo* brain uptake methods may be the most reliable evaluation of BBB penetration. However, the low-throughput, expensive, and labor-intensive characteristics make these methods inapplicable in early drug discovery stages. For these reasons,* in vitro* and* in silico* methods have been introduced. As there is no one* in vitro* model which can mimic all properties of the* in vivo* BBB, developing more reliable models remains challenging [4]. So far, a great number of* in silico* BBB models have been developed and thoroughly reviewed [2, 3, 5–9]. Because of the high complex nature of the BBB, most computational models only account for passive diffusion.

Initial studies were focusing on making correlation between BBB permeability of small set of compounds and simple descriptors and then revealed “rules of thumb.” These rough models reflect some important relationships between BBB penetration and properties of compounds but have a problem of oversimplification [10, 11]. As the accumulation of new data, various more sophisticated models were reported to predict BBB permeability. Classification models [12] which were used widely explored for distinguishing between the molecules capable of being across the BBB and those restricted to periphery. These models often developed by using the same dataset of about 1500 drugs compiled by Adenot and Lahana [13], which is the largest single homogeneous up-to-date source of qualitative data published. Some others [12, 14] distinguish molecules based on a certain log⁡*BB* threshold. However, the main problem is the threshold which is subjectively determined and not unified. Most quantitative models were developed by building QSAR models [10, 15–18]. Since different datasets and validation methods were used, it is difficult to compare the performance of these models [19]. Recently, Carpenter et al. [20] developed a new model predicting the BBB penetration using molecular dynamic simulations and received good results, providing new thread of BBB permeability prediction. Here we focused on log⁡*BB* models of BBB penetration by passive diffusion.

Various data mining methods have been employed in BBB penetration models, such as multiple linear regression [21, 22], partial least squares (PLS) regression [13, 23], recursive partitioning [23, 24], neural network [25–27], and support vector machine (SVM) [28–30]. SVM, which was originally developed by Vapnik and coworkers [31], has been extensively used and consistently achieves similar or superior performance compared to other machine learning methods [32]. Its main idea is to map data points to a high dimension space with a kernel function, and then these data points can be separated by a hyper plane.

For a successful SVM model, kernel parameters of SVM and feature subset selection are the two most important factors affecting the prediction accuracy. Various strategies have been adopted for the two problems. Grid-based algorithm is one of the most straightforward strategies for parameter optimization, which discretizes the parameters and then systematically searches every grid point to find a best combination of the parameters [33]. However, its use is limited due to the computational complexity and time-consumption. Gradient-based methods [34, 35] are also widely used, which require the kernel function and the scoring function differentiable to assess the performance of the parameters. Evolutionary method [36] has also been used and achieved promising results. As for the feature selection, genetic algorithms- (GA-) based [37–41], *F*-score based feature recursive elimination [42], and many other methods [43–47] have been employed. Most of these methods focus on feature selection or parameters optimization separately [45]. However, the choice of feature subset influences the appropriate kernel parameters and vice versa [48]. Hence the proper way seems to address the two problems simultaneously. GA [41], immune clonal algorithm (ICA) [49], and Bayesian approach [50] have been recently used for simultaneously feature selection and parameters optimization for SVM on general classification problems. In our study, GA was used to do parameter optimization and feature subset selection simultaneously, and an SVM regression model was developed for the blood-brain barrier penetration prediction.

## 2. Methods

**Figure 1:**
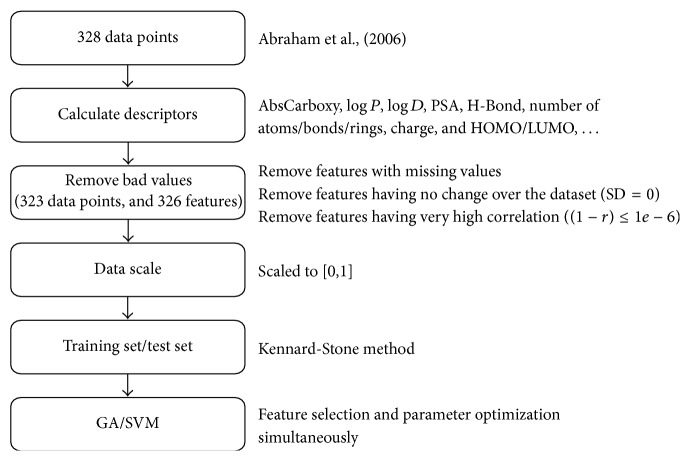
Workflow of GA/SVM model for BBB penetration prediction.

The workflow used in this study for BBB penetration prediction is illustrated in Figure 1.

### 2.1. Dataset and Molecular Descriptors

The log⁡*BB* dataset used in this study was compiled by Abraham et al. [51], which was a combination of both* in vivo* and* in vitro* data, including 302 substances (328 data points). Abraham et al. applied linear free energy relationship (LFER) to the dataset and obtained good correlation between log⁡*BB* values and LFER descriptors plus two indicator variables [51]. CODESSA [52] could not calculate descriptors for the first 5 gases ([Ar], [Kr], [Ne], [Rn], and [Xe]) of the original dataset, and they were excluded from the dataset. The final dataset contained 297 compounds (323 data points). The indicator variables of *I*_*v*_ and AbsCarboxy used in Abraham's study [51] were retained in this study. *I*_*v*_ was defined as *I*_*v*_ = 1 for the* in vitro* data and *I*_*v*_ = 0 for the* in vivo* data. AbsCarboxy was an indicator for carboxylic acid (AbsCarboxy = 1 for carboxylic acid, otherwise AbsCarboxy = 0).

The initial structures in SMILES format were imported to Marvin [53] and exported in MDL MOL format. AM1 method in AMPAC [54] was used for optimization plus frequencies and thermodynamic properties calculation. The generated output files were used by CODESSA to calculate a large number of constitutional, topological, geometrical, electrostatic, quantum-chemical, and thermodynamic descriptors. Marvin was also used to calculate some physicochemical properties of the compound, including log⁡*P*, log⁡*D*, polar surface area (PSA), polarizability, and refractivity. All these descriptors and properties were used as candidate features in later modeling.

Features with missing values or having no change across the data set were removed. If the correlation coefficient of two features is higher than a specified cutoff value (0.999999 used here), then one of them is randomly chosen and removed. The cutoff value used here is very high because very high variable correlation does not mean absence of variable complementarity [55]. A total number of 326 descriptors were left for further analysis. However, many highly correlated features have very similar physicochemical meanings. In our final analysis, similar features were put together by their physicochemical meaning, which we hope could unveil some underlying molecular properties that determine the BBB penetration.

The dataset was then split into training set and test set using the Kennard-Stone method [56], which selects a subset of representative data points uniformly distributed in the sample space [57]. At start, the Kennard-Stone method chooses the data point that is the closest to the center of the dataset measured by Euclidean distance. After that, from all remaining data points, the data point that is the furthest from those already selected is added to the training set. This process continues until the size of the training set reaches specified size. 260 data points were selected as training set and the other 63 were used as test set.

### 2.2. SVM Regression

Details about SVM regression can be found in literatures [58–60]. As in other multivariate statistical models, the performance of SVM regression depends on the combination of several parameters. In general, *C* is a regularization parameter that controls the tradeoff between training error and model complexity. If *C* is too large, the model will have a high penalty for nonseparable points and may store too many support vectors and get overfitting. If it is too small, the model may have underfitting. Parameter *ε* controls the width of the *ε*-insensitive zone, used to fit the training data. The value of *ε* can affect the number of the support vectors used to construct the regression function. The bigger *ε* is, the fewer support vectors are selected. On the other hand, bigger *ε*-values result in more flat estimates. Hence, both *C* and *ε*-values affect model complexity (but in a different way). The kernel type is another important parameter. In SVM regression, radial basis function (RBF) (1) was the most commonly used kernel function for its better generalization ability, less number of parameters, and less numerical difficulties [33] and was used in this study. Parameter *γ* in RBF controls the amplitude of the RBF kernel and therefore controls the generalization ability of SVM regression. The LIBSVM package (version 2.81) [61] was used in this study for SVM regression calculation, taking the form

(1) $$\begin{array}{c} K\left(\;x_{i},x_{j}\;\right)=\exp\left(\;-\gamma {\left\|\;x_{i}-x_{j}\;\right\|}^{2}\;\right),\;\gamma >0, \end{array}$$

where *x*_*i*_ and *x*_*j*_ are training vectors (*i* ≠ *j*, *x*_*i*_ ≠ *x*_*j*_) and *γ* is kernel parameter.

### 2.3. Genetic Algorithms

Genetic algorithms (GA) [41] are stochastic optimization and search method that mimics biological evolution as a problem-solving strategy. They are very flexible and attractive for optimization problems.

**Figure 2:**
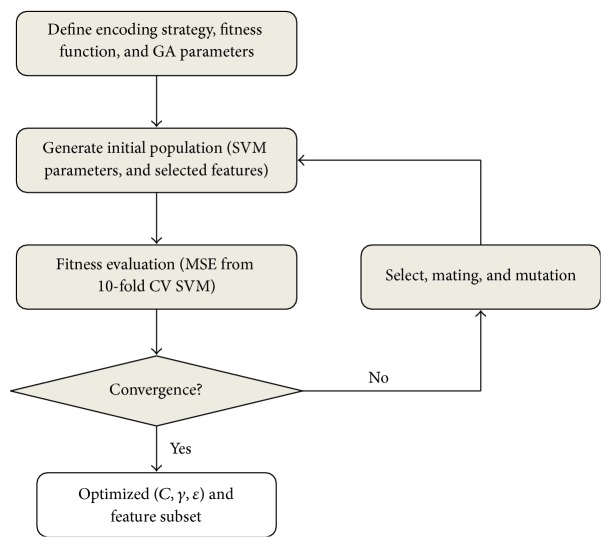
Workflow of genetic algorithms.

Given a specific problem to solve, the input to the GA is a set of potential solutions to that problem, encoded in some fashion, and a fitness function that allows each candidate to be quantitatively evaluated (Figure 2). Selection, mating, and mutation just mimic the natural process. For each generation, individuals are selected for reproduction according to their fitness values. Favorable individuals have a better chance to be selected for reproduction and the offspring have chance to mutate to keep diversity, while the unfavorable individuals are less likely to survive. After each generation, whether the evolution is converged or the termination criteria are met is checked; if yes, job is done; if not, the evolution goes into next generation. After many generations, good individuals will dominate the population, and we will get solutions that are good enough for our problem.

**Figure 3:**
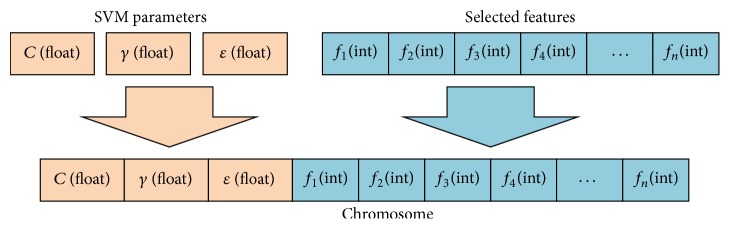
Encoding of the chromosome.

First, in order to solve a problem with GA, each individual in the population should be represented by a chromosome. In our study, since the parameter optimization and feature subset selection should be addressed simultaneously, the chromosome is a combination of parameter genes and feature gene (Figure 3), where *f*_*n*_ is an integer in the range of [1, *N*] and *N* is the number of candidate features for model construction. A chromosome represents an individual in genetic algorithms and parameters contained in chromosome could be used for SVM modeling. Left part of the chromosome is the parameter genes, of which *C*, *γ*, and *ε* all are float genes. The feature gene is an array of integers, and each integer represents a feature.

Fitness function can be seen as a ruler, which was used to quantitatively evaluate and compare each candidate. In our study, the mean squared error (MSE) of 10-fold cross validation (CV) for SVM was used as fitness function, and smaller fitness value indicated better individual. Given a training set containing *n* compounds, (*x*_1_, *y*_1_),…, (*x*_*n*_, *y*_*n*_), *x*_*i*_ is descriptor vector of compound *i* and *x*_*i*_ ∈ *R* ^*n*^. *y*_*i*_ is the log⁡*BB* value and *y*_*i*_ ∈ {−1, +1}. The objective function can be calculated by

(2) $$\begin{array}{c} f\left(\;x\;\right)=\overset{n}{\underset{i=1}{\sum }}\left(\;\alpha_{i}-\alpha_{i}^{*}\;\right)K\left(\;x_{i},x\;\right)+b. \end{array}$$

*α*_*i*_ and *α*_*i*_^*∗*^ are Lagronia factors, *K*(*x*_*i*_, *x*) is kernel of radial basis function. The MSE of 10-fold CV for SVM was calculated by

(3) $$\begin{array}{c} MSE=\frac{1}{n}\overset{n}{\underset{i=1}{\sum }}{\left(\;\hat{X_{i}}-X_{i}\;\right)}^{2}, \end{array}$$

where *n* is the number of all data points, $\hat{X_{i}}$ is the predicted value, and *X*_*i*_ is the experiment value.

Tournament selection was used as the selection strategy in GA, which selected the best 1 from 3 randomly chosen candidates. The advantage of tournament selection over roulette wheel selection is that tournament selection does not need to sort the whole population by fitness value.

**Figure 4:**
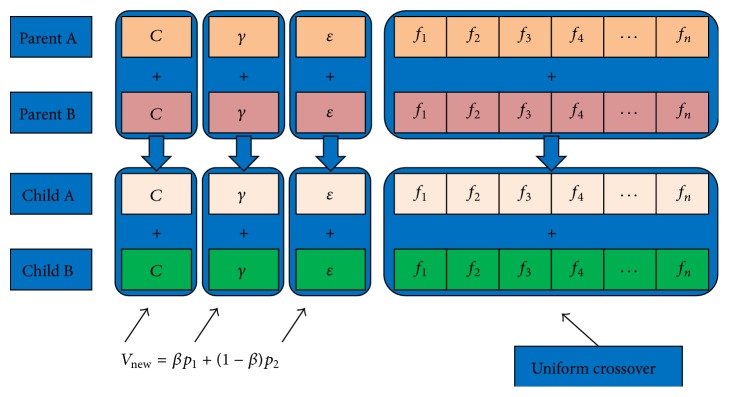
Mating strategy of GA.

Since there are different types of genes in a chromosome, different mating strategies were used for different types of genes (Figure 4):

(4) $$\begin{array}{c} V_{new}=\beta p_{1}+\left(\;1-\beta \;\right)p_{2}, \end{array}$$

where *β* uniformly distributed random number on the interval [−0.25,1.25], *p*_*n*_ is the value of parent gene, and *V*_new_ is the value of child gene.

For float genes, the new value is a linear combination of the parents (4). For feature gene, uniform crossover is used: each element of the child gene is selected randomly from the corresponding items of parents.

Again, different mutation strategies were used for different types of genes. For float genes, the values were randomly mutated upward or downward. The new value was given by

(5) $$\begin{array}{c} V_{new}=\left\{\begin{array}{cc} V-\beta \left(V-V_{min}\right) & \text{if random}\left(\right)<0.5\\ V+\beta \left(V_{max}-V\right) & \text{if random}\left(\right)\geq 0.5, \end{array}\right. \end{array}$$

where *β* was a random number distributed in [0, 1], *V* and *V*_new_ are values before and after mutation, and *V*_min_ and *V*_max_ are the minimum and maximum values allowed for a gene.

For feature gene, several points were first randomly chosen for mutation, and then a random number in [1, *N*] (*N* is the total number of features) was chosen as new feature while avoiding duplicate features. The GA was terminated when the evolution reached 1000 generations. In our pilot study (data not shown), 1000 generations were enough for the GA to converge. The other parameters for GA were as follows: population size 100, cross rate 0.8, mutation rate 0.1, elite size 2, and number of new individuals in each generation 8.

## 3. Results and Discussion

### 3.1. GA/SVM Performance

**Figure 5:**
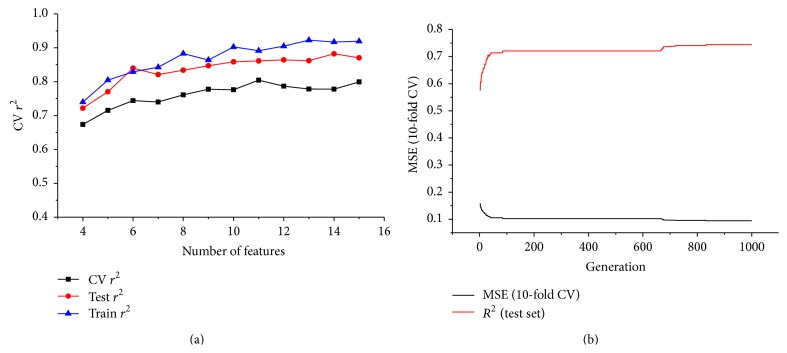
(a) Performance comparison of models with different number of features. (b) Evolution of the best 6-feature model.

GA was run with different number of features from 4 to 15. For each number of features, GA was run 50 times, and the best model was chosen for further analysis. From Table 1 and Figure 5, the overall trend of the GA showed the following: (1) the accuracy of the model increased with the number of the features; (2) the accuracy of the model on training set was better than the accuracy on the test set, which was then better than the accuracy of cross validation.

**Figure 6:**
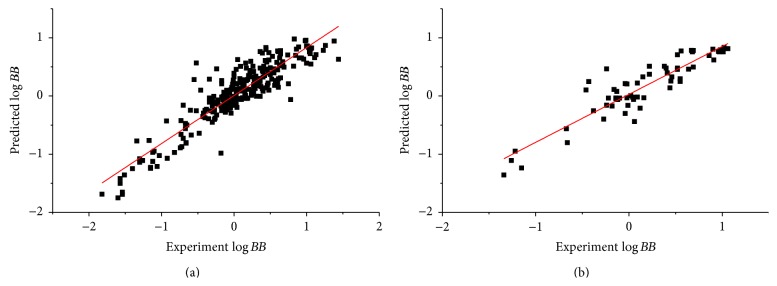
Prediction accuracy of the final model on training set (a) and test set (b).

As the feature number increases, the complexity also increases, which will often increase the probability of overfitting. A complex model is also difficult to interpret and apply in practical use, so generally speaking, we need to find a balance between the accuracy and complexity of the model. It is observed that (Table 1, Figure 5(a)) the prediction accuracy (*r* ^2^ = 0.744) of cross validation (*n* = 10) of the 6-feature model was similar to that of the Abraham's model (*r* ^2^ = 0.75) which used all 328 data points (Table 2). As the number of features increased from 6 to 15, the prediction accuracy on training set increased from 0.829 to 0.919, while the prediction accuracy on test set only slightly increased from 0.840 to 0.870 accordingly. Take all these into consideration, the 6-feature model (Table 1, Figure 5(a)) was chosen as our final model (Figure 6), of which the prediction accuracy on both test set and training set was similar (*r*_train_^2^ − *r*_test_^2^ = 0.11) and high enough (>0.82).

Figure 5(b) showed the evolution of the prediction performance of the model (MSE and *r*_test_^2^). In the first 100 generations, the MSE decreased very fast, followed with a platform stage from about 100 to 650 generations. Another decrease occurred at about 650 generations, and then the evolution became stable at about 850 generations. The models did not improve much in the last 150 generations, which may imply a convergence.

A tabular presentation of relevant studies regarding the prediction of the blood-brain distribution is shown in Table 2. These models were constructed by using different statistical learning methods, yielding different prediction capability with *R*_test_^2^ ranging from 0.5 to over 0.9. Generally, regression by SVM appears to be more robust than traditional linear approaches such as PLS and MLR, with respect to the nonlinear effects induced by multiple potentially cooperative factors governing the BBB permeability. For example, the SVM model by Golmohammadi et al. [62] yielded the highest *R*_test_^2^ on a test set containing 110 molecules. However, it should be noted that direct comparison with results from previous studies is usually inappropriate because of differences in their datasets. In this study, a combination of both* in vivo* and* in vitro* data compiled by Abraham et al. [51] was used for developing BBB prediction model, which is of high data quality and covers large chemical diversity space. In addition to the data source, kernel parameter optimization and feature selection are two crucial factors influencing the prediction accuracy of SVM models. To reduce the computational cost, most of the existing models addressed the feature selection and parameter optimization procedures separately. In this study, we used a GA scheme to perform the kernel parameter optimization and feature selection simultaneously, which is more efficient at searching the optimal feature subset space.

Abraham's model [51] is the best model that is currently publicly available. A comparison of our models with Abraham's models was shown in Table 2. In Table 2, the last 3 rows are models in our study. The same dataset was used in Abraham's research and this study, but the data set was split into different training set and test set (our model: train/test = 260/63; Abraham's model: training/test = 164/164). 7 variables were used in Abraham's model, compared to 6 in our final model. The *r* ^2^ values for training set in Abraham's 164/164 model and 328/0 model were 0.71 and 0.75, respectively, compared with 0.83 for our model. It has to be noted that the size of our training set (260) is bigger than Abraham's (164).

Our model was also compared with grid method implemented with Python toolkit (grid.py) shipped with libsvm [61] for parameter optimization. First, since grid method cannot be used to select feature subset, all 326 features were used to construct a BBB prediction model. The prediction accuracy of the training set was very high (*r*_train_^2^ = 0.97) but that of the test set was disappointing (*r*_test_^2^ = 0.55). Then we used the same feature set as our final model (6 features). The result was slightly better, but still too bad for test set prediction (*r*_train_^2^ = 0.86, *r*_test_^2^ = 0.58).

So compared with the grid-based method, our GA-based method could get better accuracy with fewer features, which suggested that GA could get much better combination of parameters and feature subset. This was also observed in other's study [48].

### 3.2. Feature Analysis

**Figure 7:**
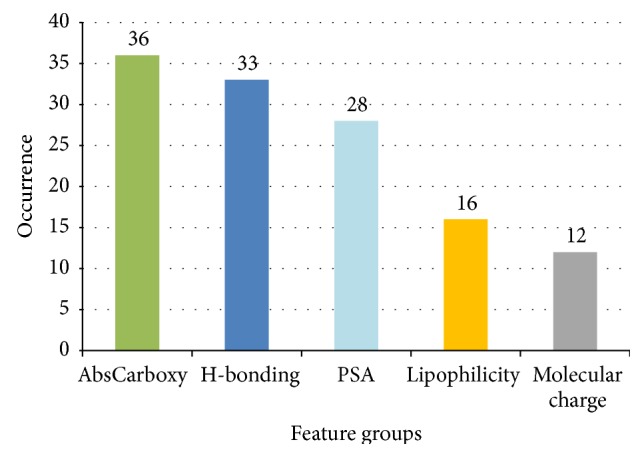
Top features for all 6-feature models (50 in all).

An examination of the descriptors used in the model could provide an insight into the molecular properties that are most relevant to BBB penetration.  Table 3 showed the features used in the final 6-feature model and their meanings. In order to explore the relative importance and the underlying molecular properties of the descriptors, the most frequently used features in all 50 6-feature models were analyzed.  Table 4 showed the top 10 most frequently used features. Interestingly, AbsCarboxy, an indicator of the existence of carboxylic acid was the most significant property. Some features with similar meaning were also found to occur in the models, such as PSA related features (M_PSA_7.4, M_PSA_7.0, and M_PSA_6.5), and H-bond related descriptors (number 267, 268, and 138). So we decided to put similar descriptors into the same group (Figure 7) to find the underlying properties affecting BBB penetration.

According to the associated molecular properties of the features, those frequently used features are categorized into 5 groups: AbsCarboxy (indicator of carboxylic acid), H-bonding (H-bonding ability, including H-bond donor/acceptor related features), PSA (molecular polar surface area related features), lipophilicity (including M_log⁡*P* and delta_log⁡*D*), and molecular charge (including charge and topological electronic index related features).

The following is observed:

(1) Interestingly, AbsCarboxy was also the most significant feature, which occurred 36 times in total 50 models. This may indicate that the carboxylic acid group plays an important role in the BBB penetration, which is consistent with the study of Abraham et al. [51].
(2) H-bonding (H-bond donor/acceptor) related surface area, polar surface area, log⁡*P* related features, and topological electronic index related features are also significant.

**Figure 8:**
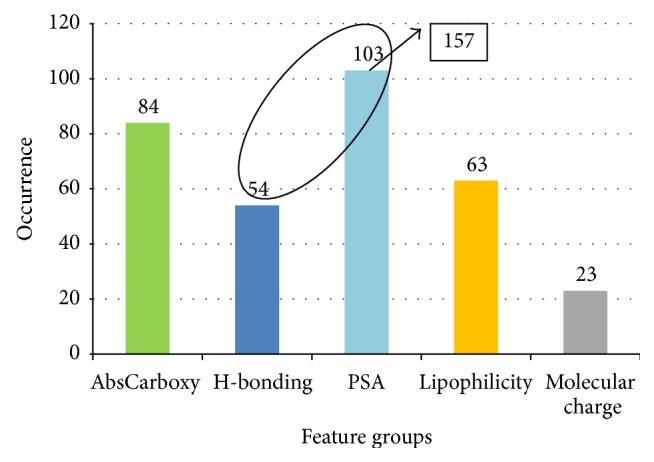
The most frequently used features for all top models.

In order to further confirm the previous finding, all top 10 models from features = 4 to 15 were further analyzed, and the result was almost the same (Table 5, Figure 8). The top 10 most frequently used feature sets were very similar. Compared to the 6-feature models, the only difference was that H-bond related feature number 267 is replaced with PSA related feature number 167.

Again, the descriptors were analyzed by group. The composites of the groups were almost the same. Given that there are 4 descriptors in PSA group, AbsCarboxy was also the most significant property, followed by PSA, lipophilicity, and H-bonding having similar occurences, then followed by molecular charge with relatively low frequencies. The high consistency suggested that these groups of features had signficant impact on the BBB penetration ability.

PSA and H-bonding descriptors are highly relevant properties: PSA is the molecular areas contributed by polar atoms (nitrogen, sulphur, oxygen, and phosphorus), and most of the time these polar atoms can be H-bond acceptor or donor. If PSA and H-bonding were merged into one group (*n* = 157), they will become the most significant property group of features.

### 3.3. Properties Relevant to BBB Penetration

#### 3.3.1. Carboxylic Acid Group

It was proposed by Abraham et al. [51] that carboxylic acid group played an important role in BBB penetration. While it was commonly believed that the most important molecular properties related to BBB penetration were H-bonding ability, lipophilicity, and molecular charge [63, 64]. However, our study confirmed Abraham's conclusion and showed that the importance of carboxylic acid group in BBB penetration could be underestimated.

In the models by Abraham et al. [51], the indicator variable of carboxylic acid group has the largest negative coefficient, indicating its importance in BBB penetration, and is consistent with observations in our model that the indicator of carboxylic acid group is the most frequently used descriptor. Zhao et al. [23] tried to classify compounds into BBB positive or BBB negative groups using H-bonding related descriptors, and the indicator of carboxylic acid group was also found to be important in their model. Furthermore, our results are consistent with the fact that basic molecules have a better BBB penetration than the acid molecules [10].

The carboxylic acid group may affect the BBB penetration through molecular charge interactions since in most cases the carboxylic acid group will exist in the ionized form carrying a negative charge. The carboxylic acid group could also affect the BBB penetration by forming H-bond with BBB and hence weaken the BBB penetration abilities of molecules.

Abraham et al. suggested that the presence of carboxylic acid group which acted to hinder BBB penetration was not only simply due to the intrinsic hydrogen bonding and polarity properties of neutral acids [51]. There were some other ways in which the carboxylic acid groups could affect the BBB penetration, such as acidic drugs which could bind to albumin [65], the ionization of the carboxylic acid groups which could increase the excess molar refraction and hydrogen bonding basicity, and finally the carboxylic acid groups which may be removed from brain by some efflux mechanism [66].

#### 3.3.2. Polar Surface Area and H-Bonding Ability

As pointed out in our previous analysis, PSA and H-bonding ability are actually two highly correlated properties. If these two groups are merged, they will be the most significant group of properties, even more significant than the carboxylic acid indicator (Figure 8). Norinder and Haeberlein [6] concluded that hydrogen bonding term is a cornerstone in BBB penetration prediction. In Zhao et al.'s study [23], PSA, AbsCarboxy, number of H-bonding donors, and positively charged form fraction at pH 7.4 were all treated as H-bonding descriptors and were found to be important in the final model.

Furthermore, almost all published models make use of molecular polar and/or H-bonding ability related descriptors, such as PSA [23, 67, 68], high-charged PSA [22], number of hydrogen donors and acceptors [23, 69], and hydrogen bond acidity/basicity [23]. And, in these models, PSA and/or H-bond ability are all negatively correlated with log⁡*BB*, which is in agreement with Abraham et al.'s study [51] in which the coefficients of the H-bond acidity and H-bond basicity are both negative.

After review of many previous works, Norinder and Haeberlein [6] proposed that if the sum of number of nitrogen and oxygen atoms (N + O) in a molecule was five or less, it had a high chance of entering the brain. As we all know, nitrogen and oxygen atoms have great impact on PSA and H-bonding. Norinder and Haeberlein [6] also concluded that BBB penetration could be increased by lowering the overall hydrogen bonding ability of a compound, such as by encouraging intramolecular hydrogen bonding. After an analysis of the CNS activity of 125 marketed drugs, van de Waterbeemd et al. [70] suggested that the upper limit for PSA in a molecule that is desired to penetrate the brain should be around 90 Å^2^, while Kelder et al. [71] analyzed the PSA distribution of 776 orally administered CNS drugs that have reached at least phase II studies and suggested that the upper limit should be 60–70 Å^2^.

Having in mind that molecules mainly cross the BBB by passive diffusion, we think it may be because molecules with strong H-bonding ability have a greater tendency to form H-bonds with the polar environment (the blood), hence weakening their ability to cross the BBB by passive diffusion.

#### 3.3.3. Lipophilicity

Lipophilicity is another property widely recognized as being important in BBB penetration, and most of the current models utilize features related to lipophilicity [22, 64, 67, 72]. Lipophilicity was thought to be positively correlated with log⁡*BB*; that is, increase the lipophilicity of a molecule will increase the BBB penetration of the molecule. Norinder and Haeberlein [6] also proposed that if log⁡*P* − (N + O) > 0, then log⁡*BB* was positive. Van de Waterbeemd et al. [70] suggested that the log⁡*D* of the molecule should be in [1, 3] for good BBB penetration. These observations are consistent with the fact that the lipid bilayer is lipophilic in nature, and lipophilic molecules could cross the BBB and get into the brain more easily than hydrophobic molecules.

### 3.4. Molecular Charge

From the viewpoint of computational chemistry, the distribution of molecular charge is a very important property that affects the molecule properties greatly. It is the uncharged form that can pass the BBB by passive diffusion. Fischer et al. [73] have shown that acid molecules with p*K*_a_ < 4 and basic molecules with p*K*_a_ > 10 could not cross the BBB by passive diffusion. Under physiological conditions (pH = 7.4), acid molecules with p*K*_a_ < 4 and basic molecules with p*K*_a_ > 10 will be ionized completely and carry net charges. As mentioned in our previous analysis, the carboxylic acid group may affect the BBB penetration through molecular charge interactions. Mahar Doan et al. [74] compared physicochemical properties of 93 CNS (*n* = 48) and non-CNS (*n* = 45) drugs and showed that 0 of 48 CNS drugs have a negative charge and CNS drugs tend to have less positive charge. These are reasonably consistent with the study of Abraham et al. [51] in which the coefficient of the carboxylic acid indicator is negative.

It has to be noted that, all these molecular properties are not independent, and they are related to each other. For example, the carboxylic acid group is related to both PSA and H-bonding ability, for the O atom in the carboxylic acid group is a polar atom and has a strong ability to form H-bonds; the carboxylic acid group, which carries charge under most conditions, is also related to molecular charge. The PSA/H-bonding ability is also correlated to molecular charge, because in many cases atoms could contribute to PSA or form H-bonds which could probably carry charges. Lipophilicity is also related to molecular charge.

We can get a conclusion that the most important properties for a molecule to penetrate BBB are carboxylic acid group, PSA/H-bonding ability, lipophilicity, and charge. BBB penetration is positively correlated with the lipophilicity and negatively correlated with the other three properties. A comparison of the physicochemical properties of 48 CNS drugs and 45 non-CNS suggested that compared to non-CNS drugs, CNS drugs tend to be more lipophilic and more rigid and have fewer hydrogen-bond donors, fewer charges, and lower PSA (<80 Å^2^) [74], which is in reasonable consistency with our finding except that the molecular flexibility is not important in our model.

There are some other properties utilized in some existing models, such as molecular weight, molecular shape, and molecular flexibility. It is suggested by van de Waterbeemd et al. [70] that molecular weight should be less than 450 for good BBB penetration, while Hou and Xu [22] suggested that the influence of molecular bulkiness would be obvious when the size of the molecule was larger than a threshold and found that molecular weight made a negative contribution to the BBB penetration when the molecular weight is greater 360. This is not widely observed in other studies. In Zhao et al.'s study [23], molecular weight was found to be not important compared to hydrogen bond properties.

Lobell et al. [68] proposed that spherical shapes have a small advantage compared with rod-like shapes with regard to BBB penetration, they attributed this to the membranes that are largely made from rod-shaped molecules and rod-like shape may become more easily trapped within membrane without exiting into the brain compartment. However, in Rose et al.'s model [75] based on electrotopological state descriptors showed that BBB penetration increased with less sketch branching. Crivori et al. [76] tried to correlate descriptors derived from 3D molecular fields and BBB penetration and concluded that the size and shape descriptors had no marked impact on BBB penetration.

Iyer et al. [67] found that increasing the solute conformational flexibility would increase log⁡*BB*, while in the study of Mahar Doan et al. [74], CNS drugs tend to be more rigid. However, the roles of molecular weight, molecular shape, and molecular flexibility in BBB penetration seem to be still unclear and not well received. Further studies are still needed.

## 4. Conclusion

In this study, we have developed a GA/SVM model for the BBB penetration prediction, which utilized GA to do kernel parameters optimization and feature selection simultaneously for SVM regression. The results showed that our method could get better performance than addressing the two problems separately. The same GA/SVM method can be extended to be used on other QSAR modeling applications.

In addition, the most important properties (carboxylic acid group, PSA/H-bond ability, lipophilicity, and molecular charge) governing the BBB penetration were illustrated through analyzing the SVM model. The carboxylic acid group and PSA/H-bond ability have the strongest effect. The existence of carboxylic acid group (AbsCarboxy), PSA/H-bonding and molecular charge is all negatively correlated with BBB penetration ability, while the lipophilicity enhances the BBB penetration ability.

The BBB penetration is a highly complex process and is a result of many cooperative effects. In order to clarify the factors that affect the BBB penetration, further efforts are needed to investigate the mechanistic nature of the BBB, and, as pointed out by Goodwin and Clark [7], the most fundamental need is for more high quality data, both* in vivo* and* in vitro*, upon which the next generation of predictive model can be built.

## Figures and Tables

**Table 1:** Performance comparison of models with different number of features.

Number of features	Training (CV = 10)		Prediction/*r* ^2^		Parameters of SVM		
	MSE	*r* ^2^	Test set	Training set	*C*	*γ*	*ε*
4	0.1197	0.674	0.722	0.740	38.8833	0.6081	0.1491
5	0.1042	0.715	0.770	0.805	16.3419	0.7973	0.2743
6	0.0945	0.744	0.840	0.829	13.3573	0.7158	0.1513
7	0.0959	0.74	0.821	0.843	34.3067	0.5218	0.1595
8	0.0883	0.761	0.834	0.883	60.9596	0.5871	0.2357
9	0.0815	0.777	0.847	0.864	3.7770	0.8764	0.1663
10	0.0823	0.776	0.858	0.903	15.2236	0.6247	0.1434
11	0.0714	0.804	0.861	0.891	5.6937	0.6531	0.1573
12	0.0780	0.787	0.864	0.905	7.2787	0.7428	0.1515
13	0.0817	0.778	0.862	0.922	4.1957	0.7791	0.1574
14	0.0812	0.778	0.882	0.917	14.8391	0.5002	0.2054
15	0.0734	0.799	0.870	0.919	4.9915	0.5231	0.1077

**Table 2:** Comparison of most relevant QSAR studies on BBB permeability.

Descriptors	*N*_train_	*N*_test_	Methods	*r*_train_^2^	Predictive accuracy on test set	Reference
Δlop *P*, log⁡*P*,and log⁡*P*_cyc_	20	—	Linear Regression	0.69	—	Young et al. [77]
Excess molar refraction, dipolarity/polarisability, H-bond acidity, and basicity Solute McGowan volume	148	30	LFER	0.75	*r*_test_^2^ = 0.73	Platts et al. [66]
Δ*G*_*W*_°	55	—	Linear Regression	0.82	—	Lombardo et al. [78]
PSA, the octanol/water partition coefficient, and the conformational flexibility	56	7	MLR	0.85	*r*_test_^2^ = 0.80	Iyer et al. [79]
CODESSA/DRAGON (482)	200	110	PLS SVM	0.83 0.97	*r*_test_^2^ = 0.81 *r*_test_^2^ = 0.96	Golmohammadi et al. [62]
Molecular (CODESSA-PRO) descriptors (5)	113	19	MLR	0.78	*r*_test_^2^ = 0.77	Katritzky et al. [15]
Molecular fragment (ISIDA) descriptors	112	19	MLR	0.90	*r*_test_^2^ = 0.83	Katritzky et al. [15]
PSA, log⁡*P*, the number of H-bond acceptors, E-state, and VSA	144	10	Combinatorial QSAR (KNN SVM)	0.91	*r*_test_^2^ = 0.8	Zhang et al. [17]
Abraham solute descriptors and indicators	328	—	LFER	0.75	—	Abraham et al. [51]
Abraham solute descriptors and indicators	164	164	LFER	0.71	*s* = 0.25, MAE = 0.20	Abraham et al. [51]
CODESSA/Marvin/indicator (6)	260	63	GA based SVM	0.83	*r*_test_^2^ = 0.84, RMSE = 0.23	This research, GA/SVM, final model *C* = 13.3573, *γ* = 0.715761, *ε* = 0.151289
CODESSA/Marvin/indicator (236)	260	63	GA based SVM	0.97	*r*_test_^2^ = 0.55, RMSE = 0.31	This research, Grid/SVM *C* = 8.0, *γ* = 0.015625, *ε* = 0.0625
CODESSA/Marvin/indicator (6)	260	63	GA based SVM	0.86	*r*_test_^2^ = 0.58, RMSE = 0.29	This research, Grid/SVM *C* = 8.0, *γ* = 1.0, *ε* = 0.125

**Table 3:** Features used in the final model.

Name	Meaning
M_log⁡*P*	log⁡*P* (Marvin)
HA_dependent_HDSA-2_[Zefirov's_PC]	H-bond donor surface area related (CODESSA)
M_PSA_7.4	PSA at pH 7.4 (Marvin)
AbsCarboxy	Carboxylic acid indicator (Abraham)
HA_dependent_HDCA-2/SQRT(TMSA)_[Zefirov's_PC]	H-bond donor charged area related (CODESSA)
Average_Complementary_Information_content_(order_0)	Topology descriptor (CODESSA)

**Table 4:** The most frequently used features for all 6-feature models^a^.

Number	Feature name	Occurrence (50 models)	Meaning
11	AbsCarboxy	36	Indicator for carboxylic acid^†^

268	ESP-FHASA_Fractional_HASA_(HASA/TMSA)_Quantum-Chemical_PC	14	H-acceptor surface area/total molecular surface area^#^

101	Topographic_electronic_index_(all_bonds)_Zefirov's_PC	12	Topological electronic index for all bonded pairs of atoms^b‡^

8	M_PSA_7.4	11	PSA at pH 7.4^c§^

267	ESP-HASA_H-acceptors_surface_area_Quantum-Chemical_PC	10	H-acceptor surface area^#^

5	delta_log⁡*D*	9	log⁡*D* (pH 6.5) − log⁡*D* (pH 7.4)^d,e∧^

7	M_PSA_7.0	9	PSA at pH 7.0^§^

138	HA_dependent_HDCA-2_[Zefirov's_PC]	9	H-donors charged surface area^#^

6	M_PSA_6.5	8	PSA at pH 6.5^§^

1	M_log⁡*P*	7	log⁡*P* ^∧^

^a^Rows with the same symbol could be categorized into the same group.

^b^Topological electronic index is a feature to characterize the distribution of molecular charge: *T* = ∑_(*i*<*j*)_^*N*_*B*_^(|*q*_*i*_ − *q*_*j*_|/*r* ^2^_*ij*_), where *q*_*i*_ is net charge on *i*th atom and *r*_*ij*_ is the distance between two bonded atoms.

^c^7.4 is the pH in blood.

^d^6.5 is the pH in intestine.

^e^ *D* is the ratio of the sum of the concentrations of all species of a compound in octanol to the sum of the concentrations of all species of the compound in water. For neutral compounds, log⁡*D* is equal to log⁡*P*.

**Table 5:** Most frequently used features for all top models (number of features range from 4 to 15)^*∗*^.

Number	Descriptor name	Occurrence (120 models)	Meaning
11	AbsCarboxy	84	Indicator for carboxylic acid^†^

5	delta_log⁡*D*	35	log⁡*D* (pH 6.5) − log⁡*D* (pH 7.4)^∧^

7	M_PSA_7.0	32	PSA at pH 7.0^§^

1	M_log⁡*P*	28	log⁡*P* ^∧^

138	HA_dependent_HDCA-2_[Zefirov's_PC]	27	H-donors charged surface area^#^

268	ESP-FHASA_Fractional_HASA_(HASA/TMSA)_Quantum-Chemical_PC	27	H-acceptor surface area/total molecular surface area^#^

6	M_PSA_6.5	25	PSA at pH 6.5^§^

167	PPSA-1_Partial_positive_surface_area_[Quantum-Chemical_PC]	25	Partial positive surface area^§^

101	Topographic_electronic_index_(all_bonds)_Zefirov's_PC	23	Topological electronic index for all bonded pairs of atoms^‡^

8	M_PSA_7.4	21	PSA at pH 7.4^§^

^*∗*^Rows with the same symbol could be categorized into the same group.
